# Untargeted Metabolomics of Meat Digests: Its Potential to Differentiate Pork Depending on the Feeding Regimen

**DOI:** 10.3390/molecules28217306

**Published:** 2023-10-27

**Authors:** Martina Cirlini, Laura Righetti, Lorenzo Del Vecchio, Elena Tonni, Luigi Lucini, Chiara Dall’Asta, Gianni Galaverna

**Affiliations:** 1 Department of Food and Drug, University of Parma, Parco Area delle Scienze 27/A, 43124 Parma, Italy; laura.righetti@wur.nl (L.R.); lorenzo.delvecchio@unipr.it (L.D.V.); elena.tonni@studenti.unipr.it (E.T.); chiara.dallasta@unipr.it (C.D.); gianni.galaverna@unipr.it (G.G.); 2 Laboratory of Organic Chemistry, Wageningen University, 6708 WE Wageningen, The Netherlands; 3 Wageningen Food Safety Research, Wageningen University & Research, 6700 AE Wageningen, The Netherlands; 4 Department for Sustainable Food Process, University Cattolica del Sacro Cuore, Via Emilia Parmense 84, 29122 Piacenza, Italy; luigi.lucini@unicatt.it; 5 Interdepartmental Center for Safety, Technologies and Innovation in Agrifood (SITEIA.PARMA), University of Parma, Parco Area delle Scienze, Padiglione 33, 43124 Parma, Italy

**Keywords:** pig meat, feeding regimen, conventional diet, polyphenols, gastrointestinal digestion, untargeted metabolomics

## Abstract

Meat quality seems to be influenced by the dietary regimes applied for animal feeding. Several research studies are aimed at improving meat quality, preserving it from oxidative processes, by the incorporation of antioxidant components in animal feeding. The main part of these studies evaluates meat quality, determining different parameters directly on meat, while few research studies take into account what may happen after meat ingestion. To address this topic, in this study, an in vitro gastrointestinal digestion protocol was applied to two different pork muscles, *longissimus dorsi* and *rectus femoris*, obtained from pigs fed with different diets. In detail, two groups of 12 animals each were subjected to either a conventional diet or a supplemented diet with extruded linseeds as a source of omega-3 fatty acids and plant extracts as a source of phenolics antioxidant compounds. The digested meat was subjected to an untargeted metabolomics approach. Several metabolites deriving from lipid and protein digestion were detected. Our untargeted approach allowed for discriminating the two different meat cuts, based on their metabolomic profiles. Nonetheless, multivariate statistics allowed clearly discriminating between samples obtained from different animal diets. In particular, the inclusion of linseeds and polyphenols in the animal diet led to a decrease in metabolites generated from oxidative degradation reactions, in comparison to the conventional diet group. In the latter, fatty acyls, fatty aldehydes and oxylipins, as well as cholesterol and vitamin D3 precursors and derivatives, could be highlighted.

## 1. Introduction

Meat quality, in particular in terms of fat composition, is affected by the animal feeding regimen. For this reason, the current trend is toward formulation of specific diets for animals, such as pigs, in order to obtain meat with specific and healthier characteristics. As an example, following the use of linseeds in feed formulation, the ratio between n-6 and n-3 polyunsaturated fatty acids, considered a risk factor for both cardiovascular disease and cancer, significantly decreases in pig meat [1]. Moreover, a different dietary regimen may influence the fatty acid composition of meat: higher percentages of saturated fatty acids, as lauric and myristic acids, were found in meat samples deriving from animals fed with a palm kernel oil-enriched diet, while higher polyunsaturated fatty acid contents were detected in meat samples obtained from pigs fed with a soybean oil-enriched diet [2]. As a consequence, the effect exerted by the animal diet on the fatty acids profile can be observed in finished products, as well. As an example, a linoleic acid-enriched diet has led to a higher content of linoleic acid in seasoned preserved pork products, as well as to an increase in conjugated linoleic acids (CLAs), which was obtained in fresh loin [3], dry-cured Parma ham [4] and culatello cured meat [5].

Meat quality, both in terms of physico-chemical composition and rheological properties, could depend indeed on many factors: environmental and growth conditions, feeding, age of animals, slaughter conditions, genetic characteristics, post-mortem treatments, manufacturing processes, etc. [6]. For instance, it has been reported that after adding vitamin E to the pig diet, the springiness, cohesiveness and adhesiveness of the backfat inner layer were positively influenced [7]. Moreover, a diet enriched with vegetable oils, such as palm kernel oil, palm oil and soybean oil, combined with a decrease in dietary proteins, positively affected the tenderness of pork loin [2].

To address the contribution of all the factors that can influence the overall quality of meat, “omics” techniques coupled with chemometric tools may be successfully used to explore such a large variability [8,9]. In particular, proteomic and genomics approaches have been applied to investigate meat authenticity and species origin, as well as the changes occurring during meat processing [10,11].

In recent times, great attention has been given to the transformation of food components once entering the human body. In this context, the use of a consensus gastrointestinal simulation assay [12] has become a golden standard in the assessment of metabolites formation, bioaccessibility and bioavailability. This approach has been already used to identify the peptide profile in meat digests [13,14,15,16]. The generated information was then used for the identification of peptides of potential interest for their biological activity [17]. However, to the best of our knowledge, the untargeted fingerprint has not yet been integrated in such a pipeline so far. This would offer a superior discriminant power and the possibility to identify different classes of metabolites within a single experiment.

Therefore, this study aims to investigate the profile of pork meat digests obtained from animals under different feeding regimens. In particular, a barley-based compound feed was used for the control group, while the same feed was enriched with extruded linseeds and plant extracts for treated pigs, in order to increase the intake of n-3 polyunsaturated fatty acids and antioxidant compounds. From the control and treated groups, two muscles—*longissimus dorsi* and *rectus femoris*—were sampled and subjected to further digestion and analysis. Results could then be related to potential positive activity in the human body and ultimately used to further finetune animal feeding and improve pork meat quality.

## 2. Results

### 2.1. Multivariate Modeling

Untargeted UHPLC-HRMS analyses were performed on the digested meat samples gained from the in vitro gastrointestinal simulated digestion process.

To perform sample classification, at first, all the chromatograms were independently aligned. This returned a primary dataset with 5728 features. The primary filtering step excluded the background peaks present in the blank digested sample. The second filtering step was performed by choosing all the molecular features present in at least 80% of the samples in one group, leaving 686 and 490 peaks for the two different diets, T1/T2, and the two meat cuts, RF/LD, comparison, respectively.

At this point, the principal components analysis (PCA) models were built to investigate metabolome patterns and, therefore, relatedness/unrelatedness between the samples. The mechanism is based on the ability of the PC model to cluster samples in an unsupervised approach, since no information on group identity is used to build the model.

**Figure 1:**
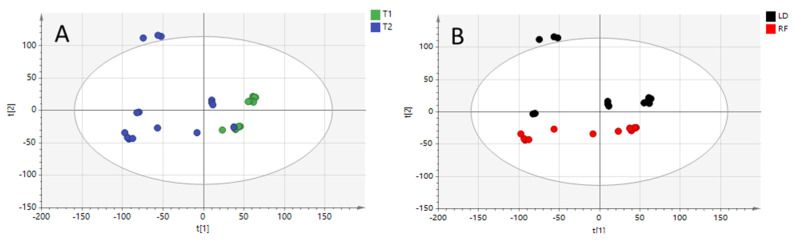
Unsupervised principal components analysis (PCA) models built from positive ionization dataset (R2X 0.639; Q2 0.44), and colored according to feed regimen (**Panel A**: T1 and T2 diets) and meet cuts (**Panel B**: longissimus dorsi “LD” and rectus femoris “RF”).

The PCA score plots obtained are depicted in Figure 1. The first three principal components (PCs) explained 57% of the total variance of the model. PCA scatter dots were colored according to the two different comparisons investigated herein. Indeed, PC1 has an influence on separating samples according to the diet (T1 and T2), while PC2 seems to better clusterize samples on the basis of the meat cuts.

Overall, 3 out of 32 samples fell outside the 95% confidence ellipse, as it is shown in the PCA score plot (Figure 1). However, investigating the Hotelling’s T2 plot, these samples were recognized as “moderate” outliers, being borderline with the 95% confidence interval. For this reason, these samples were kept in the dataset and further processed.

**Figure 2:**
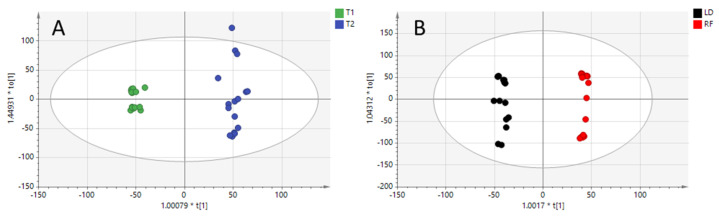
OPLS-DA model built with positive ionization data for diet regimen (R2X = 0.525, R2Y = 0.988, Q2 = 0.808) (**Panel A**: T1 and T2 diets) and for meat cuts comparison (R2X = 0.516, R2Y = 0.993, Q2 = 0.974) (**Panel B**: longissimus dorsi “LD” and rectus femoris “RF”).

At this point, two different supervised models were constructed on the basis of the different clusters observed in the PCA model. Orthogonal partial least-squares discriminant analysis (OPLS-DA) models were constructed to highlight key variables. The quality of both models was excellent (see Figure 2A,B), with prediction ability (Q2) values higher than 0.8. The spread of T2 samples depicted in Figure 2A is reasonable due to the influence of the meat cuts.

On the contrary, the OPLS-DA model built for meat cuts comparison (Figure 2B) was influenced by the two diets, with T2 and T1 samples slightly separated on the X-axis.

In addition, to avoid the risk of overfitting, each generated model was validated by a cross-validation tool, using the leave-1/3-out approach. The percentage of total correct classification was 100% for both OPLS-DA models, with all the samples correctly predicted.

### 2.2. Annotation of Statistically Significant Molecular Markers of Differentiation

Compounds annotation was achieved on the basis of the accurate mass spectra registered and using online available databases. Blank digested samples were also analyzed to exclude all the peaks present as background.

Among the assigned metabolites, classified on the basis of their chemical nature, it was possible to mainly observe lipids and peptides derivatives. The compounds having the highest discrimination potential between samples derived from the different alimentary regimes (T1 and T2) are listed in Table 1, whereas those discriminating digested samples on the basis of the different meat cuts are summarized in Table 2. In both tables, the more significant compounds are listed together with their VIP scores, using a cut-off value of >1.5.

## 3. Discussion

The applied approach allowed for differentiating meat samples on the basis of the different feeding regimens, as well as on the basis of the diverse meat cuts.

In general, both clusterizations observed are characterized by differences in small peptides profiles, mainly di- and tripeptides. This is probably due to a different proteolytic process occurring in the gastric step (GI) in the in vitro model. The composition, as well as the buffering capacity of the matrix, may actually affect protein hydrolysis in vitro. The adopted protocol [12] is indeed designed to maximize protein digestion. In detail, pepsin was added as the proteolytic enzyme during the GI phase, while pancreatin was used in the duodenal step for its proteolytic, lipolytic and amylolytic activities. No gastric lipase was added [12]. Being designed to maximize proteolysis, the protocol may suffer from slight bias when comparing different matrices. Such slight bias, although neglectable on a large scale, may be critical when di- and tripeptides are considered. Therefore, although significant in our classification model based on PCA and OPLS-DA methods, differences in small peptides between RF and LD, as well as between T1 and T2, are not discussed herein.

Concerning the effect of the diet on the metabolite profiles in digests—phospholipids, well-known constituents of cell membranes, are major contributors in the clusterization of T1 and T2 samples. They are actually more accumulated in T1 control samples, with the only exception phosphatidylglycerol PG (22:4) (Table 1). These data are in agreement with previous studies reporting that the acylation of glycerol-3-phosphate to produce phosphatidyl derivatives in animals may depend on the nutrition state of the subjects [18]. Among fatty acyls, octadecatetraenoic acid (C18:4) was significantly higher in T1 compared to T2. Being C18:4, an intermediate of the eicosanoids pathway, its accumulation can be affected by the modulation of n-3 and n-6 fatty acids in the diet. Consistently, fatty aldehydes (octadecatrienal, eicosenal and octadecenal) and two oxylipins (namely, 15(16)-epODE and 12,13-DiHOME), all deriving from an oxidative degradation of fatty acids, were mostly related to control samples. Both 15(16)-epODE and 12,13-DiHOME are two oxygenated derivatives of linoleic acid, generated from lipid peroxidation reaction [19]; thus, their lower accumulation in T2 samples is related to the higher content of antioxidant compounds in the diet.

As an additional confirmation of the higher lipid oxidation that occurred in control samples, four secosteroids involved in the vitamin D3-related pathway are more accumulated in the control than in treated samples.

The degradation of lipids by reactive oxygen species can be delayed or inhibited by the presence of antioxidant compounds, such as polyphenols. In the last years, the use of phenolic-rich plant and herbs ingredients or extracts as meat preservatives has become more and more studied to prevent or reduce lipid oxidation [20,21]. A recent study reported on the decrease in malondialdehyde and 4-hydroxy-2-nonenal, well-known lipid oxidation products potentially toxic to humans, in the digests from pork meat added with an antioxidant phytocomplex compared to control samples [21]. In our study, a similar hypothesis can be made for the different quantity of aldehydes observed in T1 and T2 digested samples. This is also in agreement with what Nieva-Echevarría et al. reported in 2017 for vegetable oils [22].

Interestingly, compounds deriving from the metabolism of cholesterol, such as azacholesterol and dehydrocholesterol, were more abundant in T1 digests than in T2. This could be ascribed to a sort of protection due to the presence of polyphenols in the alimentary regimen applied to T2 animals. Two additional compounds derived from oxidation reactions and linked to cholesterol metabolism were found in higher amount in T1 digests, dihydroxy-cholenoic acid and hydroxycholenoic acid. Cholenoic acids, pertaining to the class of steroid derivatives and more in the detail of bile acids, could be intermediates of the cholesterol metabolism and, in particular, of the synthesis of cholic and deoxycholic acids [23]. Moreover, cholenoic acids are the results of enzymatic reactions, also involved in the activation and metabolism of the vitamin D group [24]. In our case, also metabolites of D vitamins were differently accumulated in T1 and T2 groups, with dihydroxy-oxavitamin D3 and dihydroxycholecalciferol found in a higher amount in T1 digests.

Besides lipids, control samples (T1) were also characterized by a higher amount of 4-hydroxyphenylglyoxylate, deriving from the degradative oxidation of phenylalanine [25]. Among amino acid derivatives, amino-methyl-butanol was significantly lower in T2 than in T1 samples. It is an amino alcohol probably formed from α-amino acids, valine in this case, via reduction, and it can be a precursor of purines [26,27].

In regard to meat cuts, those selected for this study differ in the content of heme-iron, which is lower in LD and higher in RF. In addition, total fat content may be different for RF and LD. Data recorded for different pork meat cuts showed an average fat content of 1.54% for *rectus femoris* and 2.02% for *longissimus dorsi* [28].

Our data clearly suggest a strong involvement of the lipid fraction, and more in details of the compounds originated by the lipoxidation cascade, in the classification of meat cuts. It has been reported that a higher content of iron in RF compared to LD samples may significantly affect the oxidative degradation via Fenton’s reaction [28]. Iron, heme and non-heme may indeed catalyze the reactions that lead to the formation of reactive oxygen species, as hydroxyl radicals, as proposed by Fenton during the study of tartaric acid peroxidation. These mechanisms may, therefore, generate the cascade of reactions involved in lipid and protein oxidation in meat [29,30,31]. In this context, the addition of antioxidant compounds to animal feeding may contrast protein and lipid oxidation and subsequent meat deterioration [32,33,34], as recently demonstrated in lamb meat from animals fed with an antioxidant-enriched diet [32].

Similarly to data reported for T1 versus T2, phospholipids also contributed to the classification of meat cuts. In particular, the two groups are discriminated by the trend of several diacylglycerophosphoserins (PSs), diacylgrlycerophospholycerols (PGs) and diacylglycerophosphoinositols (PIs), likely according to fatty acyl residues distribution (Table 2). Homocarnosine and pentylbenzene, both reported in previous studies as related to the heme-iron oxidation process [35], are able to effectively discriminate RF and LD. In particular, the high-heme-iron-containing RF meat is richer in pentylbenzene and shows lower content of homocarnosine. On the other hand, homocarnosine was in a higher amount in LD digests; in the same samples, also tauroursodeoxycholic acid was more abundant than in RF samples.

Taken altogether, our results pinpointed that the use of an extruded linseeds and phenols-enriched diet leads to a reduction in pork meat of compounds related to oxidative degradation. This trends could be different for diverse meat cuts, as shown for those considered in the present study. The results suggested that the addition of antioxidants in animal feeding may lead to obtaining a higher quality meat, and may inhibit or delay oxidation processes. This may turn into a reduction of preservatives normally used in meat products as ascorbate, sodium nitrites and/or nitrates [21]. Overall, this can lead to better appreciation from consumers, who are more likely to prefer clean label food and/or preparation containing natural compounds [36].

## 4. Materials and Methods

### 4.1. Chemicals

All the salts and reagents used in the digestion protocol—potassium chloride, potassium dihydrogen phosphate, sodium bicarbonate, sodium chloride, sodium hydroxide, ammonium carbonate, magnesium chloride hexahydrate, calcium chloride dehydrate and hydrochloric acid (37% *v*/*v*)—were purchased from Sigma-Aldrich (St. Louis, MO, USA). Enzymes as α-amylase from porcine pancreas, pepsin from porcine gastric mucosa, bile extract porcine, and pancreatin from porcine pancreas were all obtained from Sigma-Aldrich (St. Louis, MO, USA). LC-grade methanol was purchased from Sigma-Aldrich (St. Louis, MO, USA), while bi-distilled water was produced in our laboratory by a Millipore Alpha Q purification system (Waters, Billerica, MA, USA).

### 4.2. Animal Feeding and Sampling

A total of 8 Italian Large White pigs were considered in this work. From about 80 kg live weight (l.w.) to slaughter (about 150 kg l.w.), the animals were allotted to two subgroups of 4 subjects each and fed two experimental diets: a standard diet for growing–finishing pigs, barley-based (T1), and a standard diet enriched with extruded linseed (5% of feed) and plant extract (natural source of polyphenols) from grape skin (3 g/kg feed; total polyphenolic content = 10.4 g/L) and oregano (2 g/kg feed; total polyphenolic content = 3.9 g/L) (T2). In particular, the linseed fatty acid composition was characterized by the presence of α-linolenic acid (54.7% of the total fatty acid composition). Moreover, the addition of linseed to animal feeding led to an increase in the α-linolenic content in the dietary fatty acid fraction from 5.2% (T1) to 25.4% (T2) [37].

At the end of the trial, the animals were slaughtered, balanced for feeding regimen, on two different days of slaughter (replicates). Two muscles were selected for the experiments: *longissimus dorsi* “LD” and *rectus femoris* “RF”. The two muscles were collected immediately after pigs slaughtering, and the meat samples were minced, maintaining the two cuts separately in order to evaluate the effect of the diet as the contribution given by a white (LD) and a red (RF) cut separately. In this way, 4 samples of LD and 4 samples of RF were finally obtained for the T1 and T2 group separately.

The meat samples were stored at −80 °C until performing gastrointestinal digestion experiments and analyses. All the simulated digestion processes were repeated twice on each meat sample, so 32 different digests were characterized.

### 4.3. In Vitro Digestion of Pork Meat Samples

The in vitro gastrointestinal digestion of pork meat samples, and the preparation of the artificial digestive juices (salivary, gastric, duodenal and bile juices), was according to Minekus et al. 2014 [12], with slight modifications. For each sample, two digestion experiments were performed.

Briefly, to simulate oral mastication, 2.5 g of minced meat were exactly weighted into a 50 mL plastic tube and digested with 0.25 mL of amylase solution (1500 U/mL) at pH = 7 on a thermostated shaker at 37 °C and 200 strokes/minute for exactly 2 min, adding 1.75 mL of salivary juice, 12.5 µL of CaCl_2_ solution and 487.5 µL of distilled water. Gastric digestion was started by adding 0.8 mL of pepsin solution (25,000 U/mL), 3.75 mL of gastric juice, 2.5 µL of CaCl_2_ solution, 0.3475 mL of distilled water and an opportune volume of HCl 1 M to reach a pH value of 3. For this step, the samples were maintained in the same conditions (37 °C and 200 strokes/minute) for 2 h. The duodenal digestion was then started using 2.5 mL of pancreatin solution (800 U/mL), 5.5 mL of the duodenal juice, 1.25 of bile juice (160 mM), 20 µL of CaCl_2_ solution, 0.655 mL of distilled water and an opportune volume of NaOH 0.1 M to reach a pH value of 7. This step was extended for 2 h under the same conditions.

At the end of the digestion process, the samples were centrifuged at 2688 g at room temperature for 10 min, and then, 2 mL of supernatant was collected and added with 200 µL of methanol in order to stop the enzymatic activity. The obtained samples were stored at −80 °C.

Before the UHPLC-HRMS analyses, 200 µL of the stock digested sample was transferred to a 1.5 mL Eppendorf tube and extracted with 400 µL of cold methanol by mixing the solution on a vortex for 1 min. Then, the mixture was centrifuged for 30 min at 16,800 g at 4 °C. The supernatant (500 µL) was finally collected and transferred to a vial. At the same time, two quality control samples (QCs) were prepared by mixing 10 µL of each sample extract. In addition to that, blank samples were prepared by subjecting 2.5 g of bi-distilled water to the same digestion protocol used for meat samples, as to the extraction procedure applied for all the other samples. Blanks, quality controls and extracted meat digested samples were then subjected to the UHPLC-HRMS analyses and statistic elaborations. All the samples were injected twice.

### 4.4. Ultra-High-Performance Liquid Chromatography–High-Resolution Mass Spectrometry

Digested samples were then screened through a hybrid quadrupole-time-of-flight mass spectrometer coupled to an UHPLC chromatographic system. A 1290 liquid chromatograph system, equipped with a binary pump and coupled to a G6550 iFunnel quadrupole-time-of-flight mass spectrometer detector (all from Agilent Technologies, Santa Clara, CA, USA), was used. Jetstream electrospray was used as the ionization source and run as previously set up for untargeted profiling [18]. Briefly, nitrogen was used as both drying (8 L/min and 330 °C) and sheath gas (10 L/min and 350 °C), the nozzle voltage was 300 V, and the capillary voltage was 3.5 kV.

Chromatographic separation was achieved in reverse-phase mode, using a Waters Zorbax eclipse plus C18 column (150 × 2.1 mm, 1.8 μm) and under a binary elution mixture, made of water (A) and methanol (B) with ammonium formate (5 mM) and 0.1% formic acid. The injection volume was 3 μL, the flow was 0.3 μL/min, and gradient elution started from 10% B to 90% B in 35 min, as previously described [25]. The mass spectrometer was operated to acquire masses in the 100–1200 *m/z* range, in positive polarity, at a rate of 0.8 spectra/s. The MS was set in “extended dynamic range” mode, with a nominal mass resolution of 30,000 FWHM. Mass accuracy was ensured by direct infusion of lock masses (121.0509 and 922.0098 *m*/*z*) through a separate electrospray.

### 4.5. Data Processing and Chemometrics Analysis

Deconvolution and compound annotation from raw data were carried out using the software Profinder B.07 (from Agilent Technologies, Santa Clara, CA, USA), based on the ‘molecular features extraction’ algorithm and the small-molecules isotopic model. Annotation was achieved based on the database exported from Metlin, Phenol-Explorer 3.6, and using the entire isotopic profile (monoisotopic mass, isotope spacing and isotope ratio), with a maximum mass accuracy of 5 ppm for each centroid mass in the isotopic cluster. Based on the strategy applied, identification was carried out according to Level 2 (putatively annotated compounds), as set out by the COSMOS Metabolomics Standards Initiative (http://cosmos-fp7.eu/msi (accessed on 10 January 2023)). Expansion of values for chromatograms extraction was set to 10 ppm, whereas single-ion identification was excluded. Data pre-processing (mass and retention time alignment, compounds filtering) was conducted in Profinder B.07. Compounds were filtered by abundance and by frequency (only those compounds with an area >10,000 counts and appearing in 80% of samples in at least one condition were considered), normalized at the 75th percentile and baselined to the median of each compound in all samples.

The raw dataset was then exported into SIMCA 13 (Umetrics, Malmo, Sweden), Pareto scaled, and the presence of outliers was investigated according to Hotelling’s T2 (i.e., the distance from the origin in the model), using 95% and 99% confidence limits for suspect and strong outliers, respectively.

The quality of the models was evaluated by the goodness-of-fit parameter (R2X), the proportion of the variance of the response variable that is explained by the model (R2Y) and the predictive ability parameter (Q2), which was calculated by a seven-round internal cross-validation of the data using a default option of the SIMCA software. R2X and R2Y represent the fraction of the variance of the X-matrix and Y-matrix, respectively, while Q2 suggests the predictive accuracy of the model. R2X, R2Y and Q2 values close to 1 indicate an excellent model, and thus, values higher than 0.5 indicate the good quality of OPLS-DA models. In order to select the most significant and reliable variables, variable importance in the projection (VIP) was used. This parameter summarizes the importance of the X-variables, both for the X- and Y-models.

In this research, VIP with the threshold >1 was used for selection of the most significant markers. To avoid the risk of overfitting, as the results found after Multivariate Data Analysis (MVDA) are sensitive to chance correlations, statistical models have to be validated. For this reason, supervised models, i.e., OPLS-DA, were validated by cross-validation, using the leave one-third out approach. The dataset was divided into three parts, and one-third of samples were excluded to build a model with the remaining two-thirds of samples. Excluded samples, one-third of samples, were then predicted by this new model, and the procedure was repeated until all samples had been predicted at least once. Each time, the percentage of correctly classified samples was calculated by generating a misclassification table.

## 5. Conclusions

The present study was focused on the application of an untargeted UHPLC-HRMS approach to evaluate the possible differences between pork meat digests belonging to two different cuts, *rectus femoris* and *longissimus dorsi*, and two different dietary regimes, a barley-based diet and one supplemented with extruded linseed and plant extracts (grape skin and oregano). To the best of our knowledge, this study represents the first attempt to classify different pork meat cuts derived from pigs fed with different diets on the basis of the metabolic profile of their corresponding digested samples. No studies, indeed, aimed to relate the metabolic composition of meat digests to meat quality, evaluating these parameters on the basis of meat derived from animals subjected to different dietary regimes.

The results obtained from UHPLC-HRMS analyses and subsequent statistical elaborations returned a clear clusterization of the digested samples on the basis of the dietary regimen applied for feeding animals. Among all the detected metabolites, lipid and protein derivatives significantly influenced sample clusterization. In particular, differences in the lipid metabolism, degradation and oxidation products were observed, such as the accumulation of fatty acyls, fatty aldehydes, oxylipins, cholesterol and vitamin D3 precursors and derivatives. The presence of these oxidative derivatives was observed especially in digests from control diet meat cuts, while the dietetic addition of linseed and polyphenols led to an inhibition of the oxidative degradation and, therefore, to a lower accumulation of oxidation products. On the other hand, different meat cut digests showed a diverse metabolic profile in terms of phospholipids, homocarnosine and pentylbenzene, relating to their different iron and lipid contents.

It is thus possible to conclude that the diet supplementation with components rich in omega 3 and polyphenols may exert a positive influence on the meat quality, leading to a reduced oxidative degradation of lipids and proteins, in respect to a conventional diet. Moreover, characterizing digested samples, rather than extracts of pork meat cuts, allowed for investigating which metabolites may enter in contact with the gastrointestinal tract when different quality meat is consumed. This research, therefore, represents a first attempt to investigate the relationship between the meat metabolites released upon digestion and the initial meat quality. More studies on different pork meat cuts, as to other kinds of meat, should be performed in this sense.

## Figures and Tables

**Table 1:** Annotation of metabolites having the highest discrimination potential between T1 and T2 (different diets), following OPLS-DA supervised modeling and VIP (Variables of Importance in Projection) analysis.

Biochemical Class	Tentative Identification	*m/z*	Pseudomolecular Ion	Elemental Formula	Higher Metabolite Intensity	VIP Score
Vitamin D3 precursors and derivatives	25-Azacholesterol	388.3572	[M+H]^+^	C26H45NO	T1	1.7
	Dehydrocholesterol	385.3456	[M+H]^+^	C27H44O	T1	1.7
	Dihydroxy-oxavitamin D3	419.3160	[M+H]^+^	C26H42O4	T1	1.9
	Dihydroxycholecalciferol	399.3248	[M+H]^+^	C27H42O2	T1	1.7
Glycerophospholipids	PA(P-18:0)	445.2691	[M+Na]^+^	C21H43O6P	T1	1.9
	PE (24:0)	580.3982	[M+H]^+^	C29H58NO8P	T1	1.8
	PG (22:4)	697.4454	[M+H-H_2_O]^+^	C38H67O10P	T2	1.9
	PE (23:0)	566.3806	[M+H]^+^	C28H56NO8P	T1	1.8
	PA(28:2)	729.5450	[M+H]^+^	C41H77O8P	T1	1.7
	PI (18:0)	583.3242	[M+H-H_2_O]^+^	C27H53O12P	T1	1.8
	PI (18:1)	581.3091	[M+H-H_2_O]^+^	C27H51O12P	T1	1.8
	PS (25:0)	620.3869	[M+H-H_2_O]^+^	C31H60NO10P	T1	1.6
	PG(35:2)	806.5916	[M+NH_4_]^+^	C43H81O10P	T1	1.7
	4-Hydroxyphenylglyoxylate	167.0341	[M+H]^+^	C8H6O4	T1	1.8
Fatty Acyls	Octadecatetraenoic acid	277.2163	[M+H]^+^	C18H28O2	T1	1.7
	15 (16)-epODE	295.2268	[M+H]^+^	C18H30O3	T1	1.8
	12,13-DiHOME	297.2433	[M+H-H_2_O]^+^	C18H34O4	T1	1.3
Fatty aldehydes	Octadecatrienal	263.2369	[M+H]^+^	C18H30O	T1	1.7
	Eicosenal	312.3260	[M+NH_4_]^+^	C20H38O	T1	1.7
	Octadecenal	284.2949	[M+NH_4_]^+^	C18H34O	T1	1.9

**Table 2:** Annotation of metabolites having the highest discrimination potential between red (RF) versus white (LD) meat, following OPLS-DA supervised modeling and VIP (Variables of Importance in Projection) analysis.

Biochemical Class	Tentative Identification	*m/z*	Pseudomolecular Ion	Elemental Formula	Higher Metabolite Intensity	VIP Value
	Homocarnosine	263.1115	[M+Na]^+^	C10H16N4O3	LD	2.0
	Pentylbenzene	149.1323	[M+H]^+^	C11H16	RF	1.1
Glycerophospholipids	PG (30:9)	799.4918	[M+H-H_2_O]^+^	C46H73O10P	RF	2.2
	PI(35:2)	871.5308	[M+Na]^+^	C44H81O13P	RF	2.0
	PI(34:0)	839.5584	[M+H]^+^	C43H83O13P	LD	1.9
	PS(36:8)	758.4370	[M+H-H_2_O]^+^	C42H66NO10P	RF	2.0
	PS (34:3)	740.4884	[M+H-H_2_O]^+^	C40H72NO10P	LD	1.9

## Data Availability

The data presented in this study are available on request from the corresponding author.

## Sample Availability

Not available.
